# Advances of the small molecule drugs regulating fibroblast-like synovial proliferation for rheumatoid arthritis

**DOI:** 10.3389/fphar.2023.1230293

**Published:** 2023-07-21

**Authors:** Yitong Tong, Xinyu Li, Qichuan Deng, Jianyou Shi, Yibin Feng, Lan Bai

**Affiliations:** ^1^ Chengdu Second People’s Hospital, Chengdu, Sichuan, China; ^2^ Department of Pharmacy, Personalized Drug Therapy Key Laboratory of Sichuan Province, Sichuan Academy of Medical Sciences and Sichuan Provincial People’s Hospital, School of Medicine, University of Electronic Science and Technology of China, Chengdu, Sichuan, China; ^3^ School of Chinese Medicine, The University of Hong Kong, Shatin, Hong Kong SAR, China

**Keywords:** rheumatoid arthritis, fibroblast-like synoviocytes, signaling pathways, small molecule drugs, natural products

## Abstract

Rheumatoid arthritis (RA) is a type of chronic autoimmune and inflammatory disease. In the pathological process of RA, the alteration of fibroblast-like synoviocyte (FLS) and its related factors is the main influence in the clinic and fundamental research. In RA, FLS exhibits a uniquely aggressive phenotype, leading to synovial hyperplasia, destruction of the cartilage and bone, and a pro-inflammatory environment in the synovial tissue for perpetuation and progression. Evidently, it is a highly promising way to target the pathological function of FLS for new anti-RA drugs. Based on this, we summed up the pathological mechanism of RA-FLS and reviewed the recent progress of small molecule drugs, including the synthetic small molecule compounds and natural products targeting RA-FLS. In the end, there were some views for further action. Compared with MAPK and NF-κB signaling pathways, the JAK/STAT signaling pathway has great potential for research as targets. A small number of synthetic small molecule compounds have entered the clinic to treat RA and are often used in combination with other drugs. Meanwhile, most natural products are currently in the experimental stage, not the clinical trial stage, such as triptolide. There is an urgent need to unremittingly develop new agents for RA.

## 1 Introduction

Rheumatoid arthritis (RA) is a type of autoimmune joint disease. It often occurs in women and the elderly. RA might affect 0.5%–1% of the global population (Zhang et al., 2022). Among the multiple factors, genetic and autoimmune along with environmental factors might be the primary causes. It shows the clinical presentation of joint pain, thickening of the synovial membrane, pannus formation, and infiltration of various inflammatory cells in the joint space, leading to the damage of the cartilage as well as bone tissue, even remarkably joint deformity and dysfunction (Smolen et al., 2018). A lot of attention is paid to the treatment of RA because it has high morbidity, might lead to disability, and has poor prognosis (Davis et al., 2012; Almutairi et al., 2021). Currently, non-steroidal anti-inflammatory drugs (NSAIDs), disease-modifying anti-rheumatic drugs (DMARDs) (synthetic or biologic agents), and glucocorticoids (Lampropoulos et al., 2015; Zhang et al., 2022) are popular in the treatment of RA. With the use of NSAIDs, the risk of cardiovascular disease might occur as well as gastrointestinal side effects, so a comprehensive evaluation is needed (O'Shea et al., 2013). DMARDs such as methotrexate (MTX), while suppressing inflammation and joint destruction, might cause nausea, anorexia, stomatitis, alopecia, myelosuppression, and even liver and pulmonary toxicity in severe cases, which requires careful monitoring. In addition, there are also problems of high expense and gastrointestinal adverse effects for DMARDs (Zhang et al., 2019). Biologic disease-modifying anti-rheumatic drugs (bDMARDs) show therapeutic effects for RA, but there are some individual differences because of different genetic backgrounds and environmental stimuli (Lampropoulos et al., 2015), and they do not cure the disease (Yamada, 2023). There is an urgent need to continuously develop new anti-RA drugs.

The synovium is considered to be a structure of connective soft-tissue membrane located in the joint cavity and the fibrocartilage, around arthrosis to provide nutrition and lubrication (Jay et al., 2000). The fibroblast-like synoviocytes (FLSs) are highly specialized mesenchymal cells found in the synovial membrane. In normal physiological regulation, FLS produces joint lubricants, for example, hyaluronic acid which nourishes the cartilage surface and shapes the synovial extracellular matrix (ECM). However, in RA, FLS exhibits a distinctive aggressive phenotype, with this aggressive behavior toward the ECM further exacerbating joint damage (Nygaard and Firestein, 2020). For this reason, one potential strategy for treating RA is the creation of medicines that target FLS (Bartok and Firestein, 2010). It is important to note that several of their monomers appear to have a positive impact on preventing arthritic synovial hyperplasia. They are mainly related to the induction of apoptosis and the inhibition of FLS proliferation. In this review, taking the state of FLS as a starting point, we summarize and discuss the literature on the small molecule drugs of FLS from PubMed, Embase, and other databases in the recent 3 years until 28 February 2023. Specific keywords used are “RA,” “FLS,” “MAPK,” “NF-κB,” “JAK/STAT,” “Wnt,” and “signaling pathways.” The small molecule drugs contain organic compounds with low molecular weights, typically ≤1000 Da. Also, these include both synthetic compounds and natural products derived mainly from plants and animals. Publications with incomplete data or conclusions and those not directly related to RA and small molecule compounds are excluded. Here, first, there is an introduction of the pathological mechanisms of RA-FLS. Second, according to the signaling pathways controlling the abnormal behavior of FLS, small molecule drugs of related pathways, especially drugs with high anti-RA-FLS potential, are analyzed in depth. Finally, we list our comments, which we hope will provide directions to developing targeted anti-rheumatic drugs for clinics.

## 2 FLS involved in the pathogenesis of RA

**FIGURE 1:**
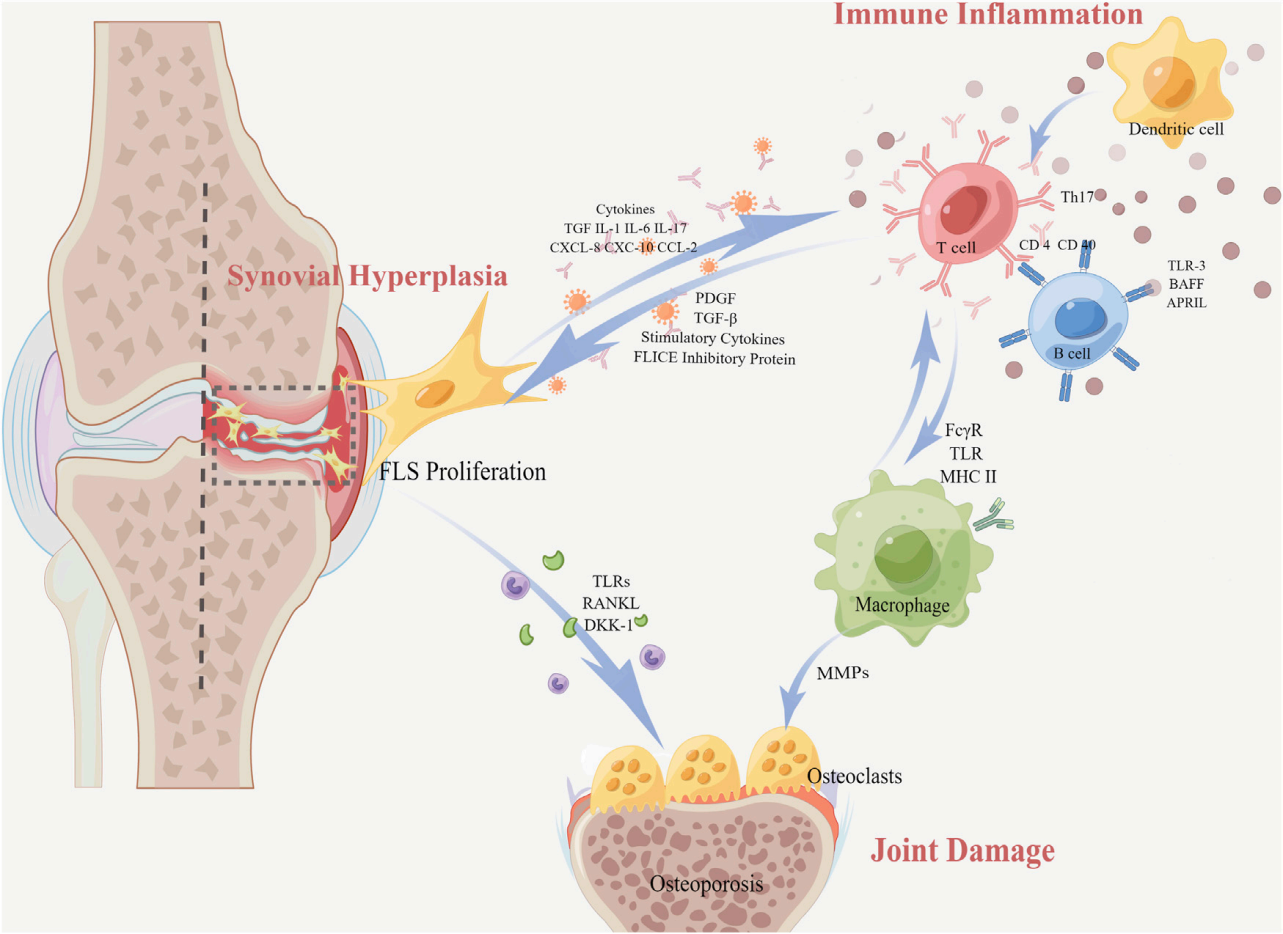
Pathological mechanisms of RA with FLS (In RA, the proliferation of FLS resulting from synovial hyperplasia releases various anti-inflammatory cytokines and growth factors. Meanwhile, the interaction between FLS and immune cells causes a transformation of regular FLS into an aggressive phenotype, resulting in an abnormal situation of T-cell and B-cell functions related to immune inflammation. Furthermore, FLS secretes pro-inflammatory cytokines into the joint space and invades the adjacent bone tissue through migration, inducing bone erosion and joint destruction. Macrophages also differentiate directly into mature osteoclasts).

In RA, FLS proliferation releases several anti-inflammatory cytokines and growth factors, among which are tumor necrosis factor (TNF), interleukin (IL) (such as IL-6, IL-1β, and IL-17), chemokines, and inflammatory enzymes [such as nitric oxide synthase (NOS) and cyclooxygenase-2 (COX-2)]. Meanwhile, it provides the inflammatory microenvironment and potentially contributes to the initiation of chronic inflammation in the preliminary stage of RA. In addition, FLS produces large amounts of receptor activator of NF-κB ligand (RANKL), vascular endothelial growth factor (VEGF), matrix metalloproteinases (MMPs), and so on, which causes synovial hyperplasia and arthritic joint destruction (Wang et al., 2012). Worse still, the activated FLS migrates to the cartilage and bone. This migration occurs not only at local sites but also through the bloodstream into distant areas and joints, destroying the cartilage, activating osteoclasts, and enhancing joint destruction in RA (Neumann et al., 2010; Hu et al., 2019). Here, we review the pathological mechanisms of RA from the three perspectives shown in Figure 1: synovial hyperplasia, joint damage, and immune inflammation.

### 2.1 Synovial hyperplasia

The synovium of RA exhibits endothelial hyperplasia and transformation into pannus tissue that destroys the articular cartilage and bone, with occasional lymphatic-like aggregates. A large number of inflammatory cytokines (IL-1β, TNF-ɑ, etc.) stimulate FLS to proliferate abnormally and exhibit anti-apoptosis. The imbalance between FLS anti-apoptotic and pro-apoptotic factors increases the number of FLS considerably, which directly leads to synovial hyperplasia. The FLS in the synovial lining layer is increased from the normal 1–3 to 10–15 cell layers (Neumann et al., 2010). The proliferated FLS develops into lymphoid-like structures, interacting with immune cells to form lymphoid organs and releasing pro-inflammatory factors and inflammatory mediators. Growth factors, such as platelet-derived growth factor (PDGF), transforming growth factor-β (TGF-β), and stimulatory cytokines in the synovial tissue, induce FLS proliferation through the activation of the signaling pathway. Along with the *in situ* proliferative capacity of FLS, the expression of anti-apoptotic molecules is also increased. The anti-apoptotic molecule FLICE inhibitory protein (FLIP) suppresses intracellular apoptosis-triggering cystatase-8, decreasing apoptosis and causing synovial proliferation (Bartok and Firestein, 2010).

### 2.2 Joint damage

Cartilage and bone destruction are hallmarks of RA. MMPs expressed by FLS degrade the chondral matrix, leading to impaired nutrient supply to the articular cartilage and tissue joint destruction.

#### 2.2.1 Chondral matrix degradation

FLS mediates the overproduction of MMPs that interrupts the joint tissue, which contains a structure abundant in collagen and facilitates FLS infestation into the cartilage surface. Mediated by pro-inflammatory cytokines and toll-like receptors (TLRs), FLS upregulates the expression of MMPs, which activate osteoclasts and directly erode the bone, causing cartilage and bone destruction. Activated osteoclasts can reduce bone mass in the periarticular bone early in the lesion, leading to osteoporosis. In addition, the extra expression of MMPs upregulates the levels of inflammatory factors and soluble mediators in the synovial tissue. Also, the factors are bound to receptors of MAPK, JAK/STAT, etc., signaling pathways, promoting and maintaining joint inflammation (Firestein, 2003).

#### 2.2.2 Bone destruction

The migration of FLS is also the process of bone destruction. Due to the cytokines, FLS can migrate into the cartilage and bone, thus exacerbating cartilage destruction (Zeng et al., 2017). FLS produces RANKL in the cartilage or bone. Then, RANKL binds to the receptor activator of NF-κB (RANK) on osteoclast precursors, inducing osteoclast differentiation, activation, and production. A large number of osteoclasts erode the surface of the adjacent articular cartilage membrane and induce bone destruction. Not only that, RA-FLS hinders the recovery process of bone erosion by hindering osteoblast activation through the secretion of dickkopf-1 (DKK-1). DKK-1 is a crucial regulatory molecule within the Wnt pathway, acting as an inhibitor of osteoclast function (Miao et al., 2013). Under specific microenvironmental conditions, macrophages can also differentiate directly into mature osteoclasts. In addition, inflammatory macrophages are a consistent source of matrix metalloproteinases, such as MMP-1, MMP-3, MMP-7, MMP-10, MMP-12, MMP-14, and MMP-25, which participate in connective tissue transformation and joint surface erosion observed in RA.

### 2.3 Immune inflammation

FLS are known to contribute significantly to RA by secreting inflammatory chemokines that interact with synovial infiltrating cells. The chemokines secreted by FLS, including, CXC motif chemokine 8 (CXCL-8), CXCL-10, and CC motif chemokine ligand 2 (CCL2), can recruit a range of immune cells into the synovial tissue. Then, the inflammatory mediators, for example, IL, TNF-α, and TGF-β1, from these immune cells in turn stimulate FLS activation, resulting in a vicious circle. Macrophages are constantly affected by inflammatory stimuli and participate in the development of chronic synovitis, bone erosion, and cartilage erosion. Macrophages express a lot of molecules on their surface, such as Fc-gamma receptors (FcγRs), TLR, and the major histocompatibility complex class II (MHCII), which in turn, regulate their own activities, activate other cells in the local microenvironment, or attract immune cells outside the joint. TNF-α, IL-6, IL-1β, IL-23, and a wide range of CXCL and CCL chemokines promote and maintain inflammation by recruiting and activating polymorphonuclear leukocytes, T cells, B cells, or monocytes.

#### 2.3.1 FLS and B cells

There is a bidirectional signaling between FLS and B cells. On one hand, FLS affects the maturation and growth of B cells by secreting cytokines. The etiology of autoimmune disorders involves both humoral immunity and B lymphocytes as significant contributors. The preservation of the B-cell pool and humoral immunity depend on the B-cell-activating factor of the TNF family (BAFF, also known as BLYS) and a proliferation-inducing ligand (APRIL). Taking TLR-3 as an example, TLR-3 triggers not only B-cell-activating BAFF but also APRIL. Both of them participate in the stimulation of B cells, thus prolonging B-cell survival (Bombardieri et al., 2011; Leah, 2011). On the other hand, B cells in turn induce the FLS inflammatory phenotype. In the FLS co-culture experiments with age-associated B cells (ABCs), ABCs induce FLS phenotype excitation through TNF-α inducing the activation of ERK1/2 and JAK-STAT1 signaling pathways, consequently promoting the persistence of RA (Qin et al., 2022).

#### 2.3.2 FLS and T cells

T-cell infiltration and excessive proliferation of FLS are significantly upregulated in RA patients. Both interact during RA inflammation to perpetuate inflammation. RA-FLS can present peptides of inflammatory antigens to antigen-specific T cells, contributing to the auto-reactive immune response in RA (Tran et al., 2007). Then, FLS expresses adhesion molecules, transmitting signals to CD4 T cells, such as vascular cell adhesion molecule-1 (VCAM-1) and intercellular cell adhesion molecule-1 (ICAM-1). Finally, these adhesive molecules interact with integrins, for instance, lymphocyte function-associated antigen 1 (LFA-1), resulting in CD4 T-cell proliferation and IL-17 secretion and exacerbation of the inflammatory response (Mori et al., 2017). At the same time, macrophages express MHCII as antigen-presenting cells, thereby participating in the activation and recruitment of pathogenic T cells. So, there is also an interaction between T cells and FLS (Tran et al., 2008; Tu et al., 2022).

To sum up, FLS can secrete pro-inflammatory cytokines such as TNF-α, IL-1β, IL-6, and MMP, in the joint space of RA patients and invade the adjacent bone tissue through migration, inducing bone erosion and joint destruction. The interaction between FLS and immune cells causes a transformation of regular FLS into an aggressive phenotype, resulting in abnormal T- and B-cell functions. Also, our body gradually loses its normal immune regulatory and protective ability (Ding et al., 2023). It is evident that FLS is the central effector cell in the pathogenesis. Given that there is no effective treatment targeted at FLS, the inhibition FLS-mediated pro-inflammatory response and subsequent tissue destruction seems to be a feasible strategy for RA (Nygaard and Firestein, 2020). In the next part, we summarize the results in the recent 3 years of small molecule drugs targeted at FLS.

## 3 Small molecule drugs regulating FLS

**FIGURE 2:**
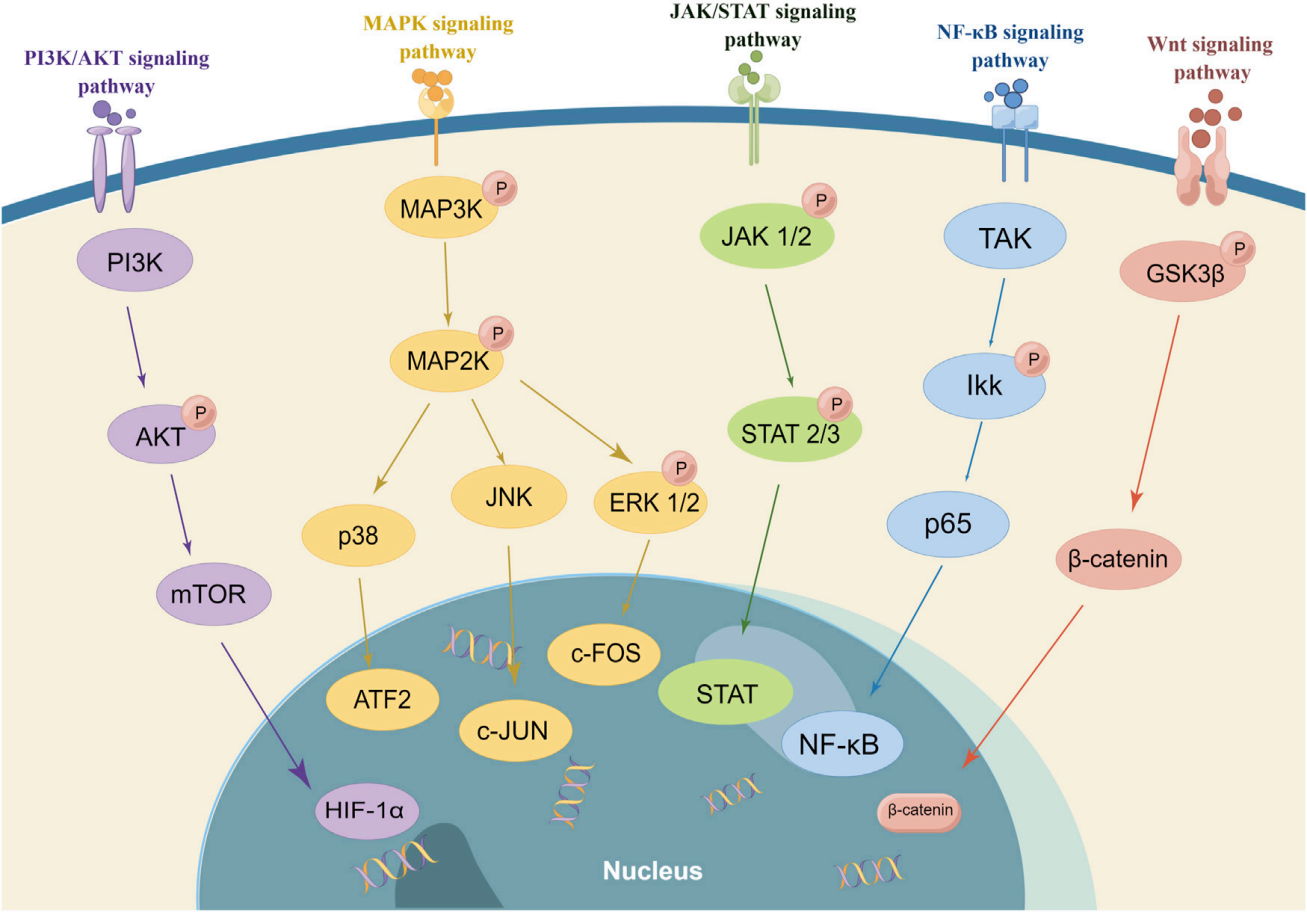
Signaling pathway regulating FLS (In RA, targeting signal transduction pathways is an emerging treatment option. The small molecule compounds and natural products interact with FLS in the different signaling pathways. There are mainly MAPK, NF-κB, JAK/STAT, PI3K/Akt, and Wnt signaling pathways. It is important to note that the majority of drugs affected numerous signaling pathways and multiple targets).

In the previous sections, we have clarified that RA-FLS are activated by multiple cytokines involved in the activation of FLS. Targeted pathways of FLS might simultaneously block multiple signaling of cytokine receptors, inhibiting the activation, proliferation, and invasion of FLS and, thus, significantly controlling RA synovial inflammation and joint damage (Mavers et al., 2009; Wendling et al., 2010; Pan et al., 2016). Despite significant breakthroughs in RA therapy, many people with RA have persistent disease. The current RA therapy plans emphasize reducing T-cell and B-cell activity as well as cytokine signaling (Mahmoud et al., 2022). In RA, targeting signal transduction pathways is an emerging treatment option. According to the signaling pathway interacted with FLS, there are mainly MAPK, NF-κB, JAK/STAT, PI3K/Akt, and Wnt signaling pathways in Figure 2. So, we present the drugs’ research progress which regulates FLS function on the signaling pathways, including the small molecule compounds and natural products. It is aimed to explore promising novel drug development directions and broaden the path of novel targeted FLS.

### 3.1 Small molecule drugs targeting MAPK regulating FLS

The MAPK signaling pathway is associated with various kinases, such as P38, c-Jun N-terminal kinase (JNK), and extracellular regulated protein kinases (ERKs), which are involved in the proliferation, apoptosis, and migration of FLS, with the addition of cytokine secretion (Harigai et al., 2004; Tang et al., 2019). ERK is involved in the secretion of certain cytokines and cell proliferation and differentiation through the regulation of B-cell lymphoma 2 (Bcl-2). JNK decreases proteoglycan synthesis and enhances MMP-13 synthesis, which are necessary for bone deterioration and joint inflammation. p38 is associated with the cytokine secretion of MMP. Through inhibiting p38, MMP reduces cartilage degradation and inhibits osteoclast formation. Additionally, the MAPK pathway contributes to the FLS’s increase in TNF-α expression, amplifying inflammatory signals, inducing FLS proliferation, aggravating inflammation, and damaging joints (Zuo et al., 2015; Kadkhoda et al., 2016). An increasing number of studies have shown that the MAPK pathway is activated in immune and autoimmune response conditions, regulating the cell responses of division, differentiation, apoptosis, inflammation, and stress and also participating in the activation of FLS (Müller-Ladner et al., 2007; Bustamante et al., 2017). In addition, MAPK activates downstream transcription factors that promote synovial cell proliferation and chondrocyte apoptosis. It also leads to high expression of multiple MMPs in synovial cells and chondrocytes and overhydrolysis of the extracellular matrix, resulting in joint damage. Therefore, MAPK is one of the most studied targets to inhibit RA-FLS (Wang et al., 2010).

**FIGURE 3:**
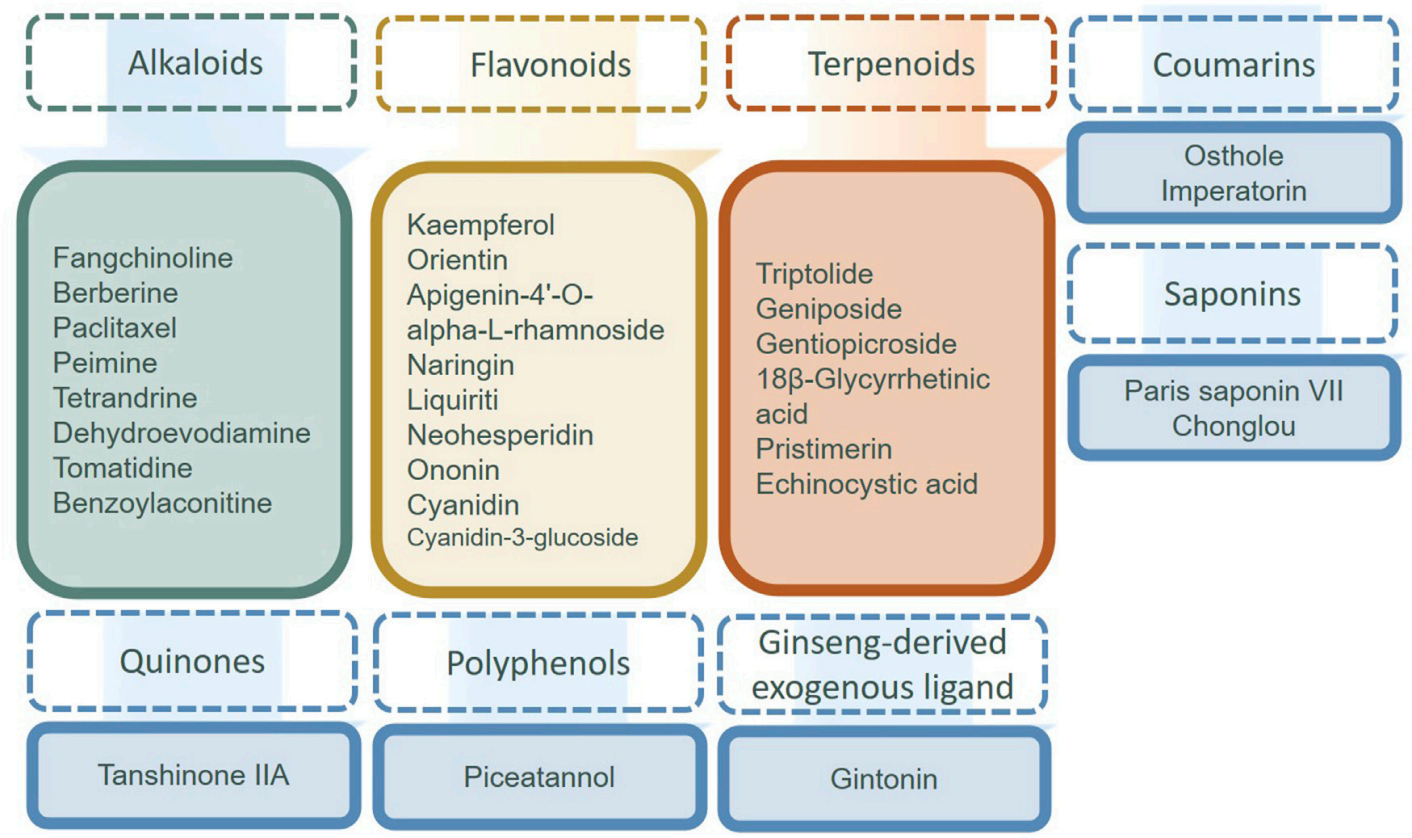
Natural products targeting MAPK regulating FLS.

Here, we review the synthetic small molecule compounds and natural products in the recent 3 years targeted to MAPK for FLS in Table 1, and the natural products regulating MAPK are shown in Figure 3. It is important to note that the majority of drugs affected numerous signaling pathways and multiple targets. As an MAPK downstream effector, p38 is considered a possible target for RA, but only few p38 inhibitors have been tested in humans. Tacrolimus as a macrolide calcineurin inhibitor immunosuppressant drug decreased the production of angiopoietin-1 (Ang1), tyrosine-protein kinase receptor (Tie-2), and VEGF in human FLS by preventing the activation of the IL-1β-mediated JNK and p38 MAPK pathways. Sugiura et al.’s (2020) study was very interesting. They found that glycogen synthase kinase 3 (GSK-3) inhibitors significantly reduced synovial fibroblast migration after 72 h and decreased Akt phosphorylation [Ser (473)] after 48 h *in vitro*, which might have therapeutic efficacy targeting the invasion and migration of synovial fibroblasts. Also, 3′3-diindolylmethane exhibited the possibility of anti-RA-FLS activitiy *in vivo* and *in vitro* (Du et al., 2019). The small molecule compounds reported in recent years that could alter FLS *in vivo* and *in vitro* were elutriated extirpate, dasatinib, 4-phenylbutyric acid, and 3-(4-hydroxy-3-methoxy-phenyl)-1-3-[1]-phenyl-propenone. Unfortunately, these medications are still in the laboratory stage. Because of their poor performance, p38 inhibitors have limited efficacy in RA treatment. Also, blocking p38’s downstream had a compensatory effect on other kinases, so alternative options for p38 have been progressively explored (Guma et al., 2012). Regulation of MAPK kinases upstream of p38, the human mitogen-activated protein kinase kinase (MKK), such as MKK6 and MKK1, could selectively block the production of MMPs and pro-inflammatory cytokines in FLS (Hammaker et al., 2012). In addition, ubiquitin D might be considered a possible therapeutic target for RA-FLS (Chen et al., 2023).

**TABLE 1:** Small molecule drugs targeting MAPK regulating FLS.

Name	Source	Targets/signaling pathways	Estimate	References
The synthetic small molecule compounds				
GSK-3 inhibitors (6-bromoindirubin-3′-oxime and thiadiazolidinone-8)	Serine/threonine protein kinase	JNK, p38, NF-κB	Experimental: NF-κB ↓	Kwon et al. (2014); Sugiura et al. (2020)
			The phosphorylated JNK, c-Jun, ATF-2, p38 ↓	
			IL-6 ↓	
			IL-10 ↑	
Tacrolimus	Macrolide antibiotics from *Streptomyces*	JNK, p38	Clinical: showed higher retention rates combined with bDMARDs	Choe et al. (2012); Kaneko et al. (2021); Terabe et al. (2023)
			Adverse events stable in long-term observation	
			Effective with acceptable safety	
			Experimental: the expressions of Ang-1, Tie-2, VEGF ↓	
3′3-Diindolylmethane	The main product of indole-3-carbinol oligomerization catalyzed by acid	p38, JNK, Akt, mTOR	Experimental: proliferation, migration, and invasion of RA-FLS *in vitro* ↓	Du et al. (2019)
			MMP-2, MMP-3, MMP-8, and MMP-9 ↓ p-p38, JNK ↓	
			Akt, mTOR ↓	
			Pro-inflammatory cytokines and arthritis severity in mice ↓	
Telotristat etiprate	A tryptophan hydroxylase inhibitor	MAPK	Experimental: migration and invasion of RA-FLS *in vitro* ↓	Zhang et al. (2023)
			Targeting LGALS3	
Dasatinib	A Src kinase inhibitor	MAPK, STATs	Experimental: Src, Fyn, MAPK, STATs ↓	Yalcin Kehribar et al. (2021); Min et al. (2023)
			MMP-1, MMP-3, MMP-13 in FLS ↓	
4-Phenylbutyric acid	An HDAC inhibitor	MAPK, NF-κB	Experimental: p-MAPK, p-NF-κB ↓	Choi et al. (2021)
			MMP-1, MMP-3, COX-2 ↓	
			Endoplasmic reticulum stress ↓	
3-(4-Hydroxy-3-methoxy-phenyl)-1-3-[1]-phenyl-propenone	A benzylideneacetophenone derivative	MAPK	Experimental: IL-8, IL-6, PGE (2) ↓	Sur et al. (2020)
			Reducing the inflammation in the knee joints in C/K-arthritic rats	
The natural products				
Fangchinoline	A bisbenzylisoquinoline alkaloid from *Stephania tetrandra*	MAPK, NF-κB	Experimental: inflammatory cytokine secretion and ROS in human FLS ↓	Villa et al. (2020)
			Phosphorylation of the MAPK and NF-κB pathway in human FLS ↓	
Berberine	An alkaloid from *Coptis chinensis*	PI3K/Akt, Wnt, RAS/MAPK/FOXO/HIF-1	Clinical: no indication for treatment of RA	Wang et al. (2019); Shen et al. (2020); Sujitha et al. (2020); Li et al. (2023); Li et al. (2023)
			Experimental: LRP5 protein ↓	
			β-Catenin transcription ↓ p38/ERK ↓	
			Proliferation and adhesion of FLS ↓	
			MMP-1, MMP-3, RANKL, TNF-α ↓	
Paclitaxel	An alkaloid from *Taxus chinensis*	MAPK, Akt/mTOR	Clinical: no indication for treatment of RA	Chen et al. (2021)
			Experimental: FLS migration dose dependently ↓	
			IL-6, IL-8, RANKL ↓	
			MMP-8, MMP-9 gene transcription ↓ p-ERK1/2 ↓	
			p-JNK ↓	
			Akt, p70S6K, 4EBP1, HIF-1α ↓	
Peimine	A steroidal alkaloid from *Fritillaria*	ERK, JNK, p38	Experimental: TNF-α induced destructive behaviors in MAPK for FLS↓	Zhou et al. (2022)
			RANKL-induced osteoclast formation ↓	
			Bone-resorption function ↓	
Tetrandrine	An alkaloid from *Stephania tetrandra* root	NF-κB, Ca_2_ (+), PI3K/Akt, MAPK	Experimental: Rac1, Cdc42, RhoA ↓	Lv et al. (2015); Zhong et al. (2019)
			MMP-2/9, F-actin, FAK↓	
			RANKL-induced osteoclastogenesis ↓	
Dehydroevodiamine	A quinazoline alkaloid from Evodiae Fructus	MAPK	Experimental: pro-inflammatory factors in AIA rats ↓	Dai et al. (2022)
			MMP-1, MMP-3 ↓ p-p38, p-JNK, and p-ERK ↓	
Tomatidine	A steroidal alkaloid from the Solanaceae family	MAPK, NF-κB	Experimental: proliferation and migration of FLS ↓	Yu et al. (2021)
			Synovial inflammation and joint destruction in CIA rats ↑	
			IL-1β, IL-6, TNF-α ↓	
			MMP-9, RANKL ↓	
Benzoylaconitine	An alkaloid from *Aconitum*	MAPK, Akt, NF-κB	Experimental: IL-6, IL-8 ↓	Yu et al. (2020)
			MAPK, p-Akt ↓	
			Degradation of IκB α↓ p-p65 and nuclear transposition ↓	
Kaempferol	A flavonoid from *Kaempferol galanga* L.	ERK-1/2, p38, JNK, NF-κB	Experimental: MAPK activation ↓, instead of altering TNF-α receptor activation	Yoon et al. (2013); Pan et al. (2018)
			Phosphorylation of ERK-1/2, p38, JNK ↓	
			NF-κB ↓	
Orientin	A flavonoid from *P. orientale*	p38, ERK	Experimental: viability, migration as well as invasion of FLS ↓	Ji and Xu (2022)
			TNFα-induced inflammatory makers ↓	
Apigenin-4′-O-alpha-L-rhamnoside	A flavonoid from apigenin derivative	MAPK	Experimental: migration of FLS ↓	Cao et al. (2022)
			MMP-1, MMP3, RANKL, TNF-α ↓	
			MAPK1, HRAS, ATF-2, p38, JNK ↓	
Naringin	A flavonoid from citrus fruits	PI3K/Akt, ERK	Experimental: inflammation, MMPs ↓	Aihaiti et al. (2021)
			Apoptosis of FLS↑ the activation of caspase-3 ↑	
			Bax/Bcl-2 ↑ p- Akt, p-ERK ↓	
Liquiritin	A flavonoid from the roots of *Glycyrrhiza uralensis*	JNK, P38	Experimental: FLS proliferation ↓	Zhai et al. (2019)
			DNA fragmentation in the nucleus ↑	
			Altering the potential of the mitochondrial membrane	
			Bcl-2/Bax ratio ↓	
			VEGF ↓ p-JNK, p-p38 ↓	
Neohesperidin	A flavanone glycoside from citrus fruits	MAPK	Experimental: IL-1β, IL-6, IL-8, TNF-α, MMP-3, MMP-9 and MMP-13 in FLSs ↓	Wang et al. (2021)
			MAPK ↓	
			ROS induced by TNF-α↓	
Ononin	An isoflavone glycoside from the fruit of *Cnidium monnieri* (L.) cusson	NF-κB, MAPK	Experimental: TNF-α mediated cells viability of FLS and MH7A ↓	Meng et al. (2021)
			Cell apoptosis↑	
			IL-1β, IL-6 ↓	
Cyanidin	An anthocyanidin from grapes, bilberry, blackberry, etc.	p38, STAT-3	Experimental: IL-17A induced the migration of monocytes from AA rats ↓	Samarpita and Rasool (2021); Samarpita et al. (2020)
			HSP27, CCR7, CXCR4 ↓	
			RANKL ↓	
			OPG ↑ p38 MAPK ↓	
Cyanidin-3-glucoside	An anthocyanin from berries	p38, ERK and JNK, NF-κB	Experimental: TNF-α, IL-1β, IL-6 ↓ p65 ↓	Sun and Li (2018)
			Phosphorylation of IκBα, p38, ERK, JNK ↓	
Paris saponin VII Chonglou	A steroidal saponin from *Trillium tschonoskii* Maxim.	JNK, p38	Experimental: FLS invasion via managing the mitochondrial apoptosis, MAPK pathway	Meng et al. (2021)
			Improving histopathological changes	
			TNF-α, IL-1β, IL-6 ↓	
			Modulating the expressions of apoptosis proteins in AIA rats	
Gintonin	A ginseng-derived exogenous ligand of lysophosphatidic acid	MAPK, NF-κB	Experimental: iNOS, IL-6, TNF-α, COX-2↓	Kim et al. (2021); Kim et al. (2021)
			NF-κB/p65 into the nucleus ↓	
Triptolide	An epoxide diterpene lactone from *Tripterygium wilfordii* Hook F.	JNK, MAPK8, PI3K/Akt	Experimental: p-JNK ↓	Yang et al. (2016); Xie et al. (2019); Song et al. (2020)
			The polymerization of F-actin ↓	
			The activation of MMP-9 ↓	
			Activating autophagy	
Geniposide	An iridoid glycoside from *Gardenia jasminoides* Ellis fruit	JNK, ERK1/2 and p38; PI3K; Akt	Experimental: proliferation of FLS ↓	Li et al. (2018); Bu et al. (2022)
			IFN-γ, IL-17 ↓	
			IL-4, TGFβ1↑ p-JNK, p-ERK1/2, p-p38 ↓	
			p-PI3K, p-Akt ↑	
Gentiopicroside	A secoiridoid glycoside from *Gentiana macrophylla* Pall.	CD147, p38, NF-κB	Experimental: proliferation of FLS ↓	Jia et al. (2022)
			MMP secretion↓	
			Regulating the CD147/p38/NF-κB pathway, p38, IkκB α, and p65 ↓	
18β-Glycyrrhetinic acid	A triterpene glycoside from *Glycyrrhiza*	MAPK, NF-κB	Experimental: IL-1β, IL-6, COX-2 in MH7A ↓	Feng et al. (2021)
			Cell viability	
			Cell apoptosis and G1 phase cell cycle arrest *in vitro* ↑	
			FOXO3 ↑	
			Liver damage caused by collagen or MTX *in vivo*↓	
			Inflammation and proliferation in FLS ↓	
Pristimerin	A triterpenoid from Celastraceae and Hippocrateaceae families	MAPK/Erk1/2, PI3K/Akt	Experimental: viability and migration of FLS ↓	Lv et al. (2022)
			TNF-α, NO, p-Akt, p-ERK ↓	
Echinocystic acid	A pentacyclic triterpene from *Gleditsia sinensis*	MAPK, NF-κB	Experimental: arthritis symptoms in SKG mice ↓	Cheng et al. (2022)
			TNF-α, IL -6, IL-1β ↓	
			P-STAT3 ↓	
			MAPK, NF-κB	
Osthole	A coumarin from *Cnidium monnieri* and *Angelica pubescens*	NF-κB, MAPK	Experimental: IL-1β, TNF-α, IL-6 ↓	Xu et al. (2018); Lin et al. (2023)
			Proliferation and migration ↓	
			TGM2/Myc/WTAP-positive feedback circuit ↓	
Imperatorin	A coumarin from Umbelliferae	p38, ERK NF-κB	Experimental: proliferation and migration of FLS ↓	Lin et al. (2022)
			TNF-α, IL-6, and IL-8 ↓ p38, ERK ↓	
			p-IκBα ↓	
Tanshinone IIA	A diterpene quinone from *Salvia miltiorrhiza* Bunge	MAPK, Akt/mTOR, HIF-1, and NF-κB	Experimental: FLS proliferation, migration, infiltration time, and dose dependently ↓	Du et al. (2020)
			MMPs, pro-inflammatory factors ↓	
Piceatannol	A derivative of resveratrol	MAPK, NF-κB	Experimental: Bax, cleaved caspase-3 ↑	Gao et al. (2022)
			PGE2, IL-6, IL-1β↓	
			COX-2 ↓	
			MMP-3, MMP-13 ↓	
			MAPK, NF-κB ↓	

↓: suppress, downregulate, inhibit, block, prevent, reduce, decrease; ↑: promote, upregulate, active, increase. mTOR, mammalian target of rapamycin; NFATc1, c-Fos and nuclear factor of activated T cells c1; ATF2, activating transcription factor-2; PGE2, prostaglandin E2; ROS, reactive oxygen species; HIF1, hypoxia-inducible factor 1; CIA, collagen-induced arthritis; IκB, inhibitor of κB; Bcl-2, B-cell lymphoma 2; Bax, Bcl-2-associated X; AA, adjuvant-induced arthritic; OPG, osteoprotegerin; MEKK, mitogen-activated protein kinase kinase; IKK, IκB kinase; TGM2, transglutaminase 2.

In natural products in Table 1 and Figure 3, alkaloids and flavonoids were more frequently reported and studied for their effects on the MAPK signaling pathway of FLS. Other categories, such as iridoids and saponins, were also found to have an impact. It is well known that flavonoids possess anti-oxidant and anti-inflammatory properties. Flavonoids can inhibit the inflammatory response and reduce the symptoms of inflammation while scavenging free radicals, reducing oxidative stress, and protecting cells from oxidative damage. Flavonoids usually inhibit FLS proliferation, migration, and invasion by inhibiting p38 and JNK. To our surprise, alkaloids also showed up significantly in the treatment of FLS. Preparations of berberine and paclitaxel were available for clinical use, but they have no indication for the treatment of RA.

Triptolide and tetrandrine from *Tripterygium wilfordii* Hook F. and *Stephania tetrandra* root, respectively, have anti-rheumatic effects in the classic sense. Tripterygium glycoside preparations have been clinically used for the treatment of RA. As the representative, we concentrate on triptolide, which has been studied more and has been proven to have multiple signaling pathways. The treatment with triptolide decreased the expression of phosphorylated JNK that TNF-α-produced, but it had no effect on the expression of phosphorylated p38 or ERK (Yang et al., 2016) and reduced FLS migration and invasion by targeting the JNK/MAPK signaling pathway (Tang et al., 2020). Triptolide dramatically increased the p-Akt/Akt ratio, and inhibiting the PI3K/Akt signaling pathway in MH7A cells caused autophagy to be triggered, indicating that triptolide repressed autophagy via activating p-Akt/Akt (Xie et al., 2019). Other natural products, such as Paris saponin VII/Chonglou, geniposide, and gentiopicroside, shown in Table 1, also have the potential to regulate FLS against RA. However, it is currently in the experimental stage.

### 3.2 Small molecule drugs targeting NF-κB regulating FLS

As a major signaling transcription factor, NF-κB contributes to synovial inflammation, proliferation, and decay in bones in RA and regulates inflammatory gene expression and cell proliferation. Both innate and adaptive immune cells include NF-B, which is a key mediator of the stimulation of pro-inflammatory genes (Liu et al., 2017). In a normal situation, NF-κB is bound to its repressor protein IκB and not activated. The nuclear-localization sequence (NLS) that belongs to NF-κB is covered by the IκB unable to undergo nuclear translocation. However, in RA due to the activators (TNF-α, IL-17, etc.), IκB is phosphorylated, ubiquitinated by IκB kinase, and eventually degraded by the enzyme, releasing NF-κB. Following that, NF-κB p65 enters the nucleus and combines with target genes (Aupperle et al., 1999). The production of inflammatory mediators such as TNF-α, COX-2, and IL-1β increases as a result of this nuclear translocation in the synovium. Those activated sustaining states lead to massive abnormal activation of FLS (Saravanan et al., 2014). NF-κB p65 regulates apoptosis and inhibits protein expression, which has an antagonistic effect on apoptosis in FLS (Kadkhoda et al., 2016), leading to synovial hyperplasia and aggravating joint destruction (Yin et al., 2015). In addition, p38 mediates IκB phosphorylation, which is involved in regulating NF-κB activation (Carter et al., 1999; Kaminska, 2005).

**FIGURE 4:**
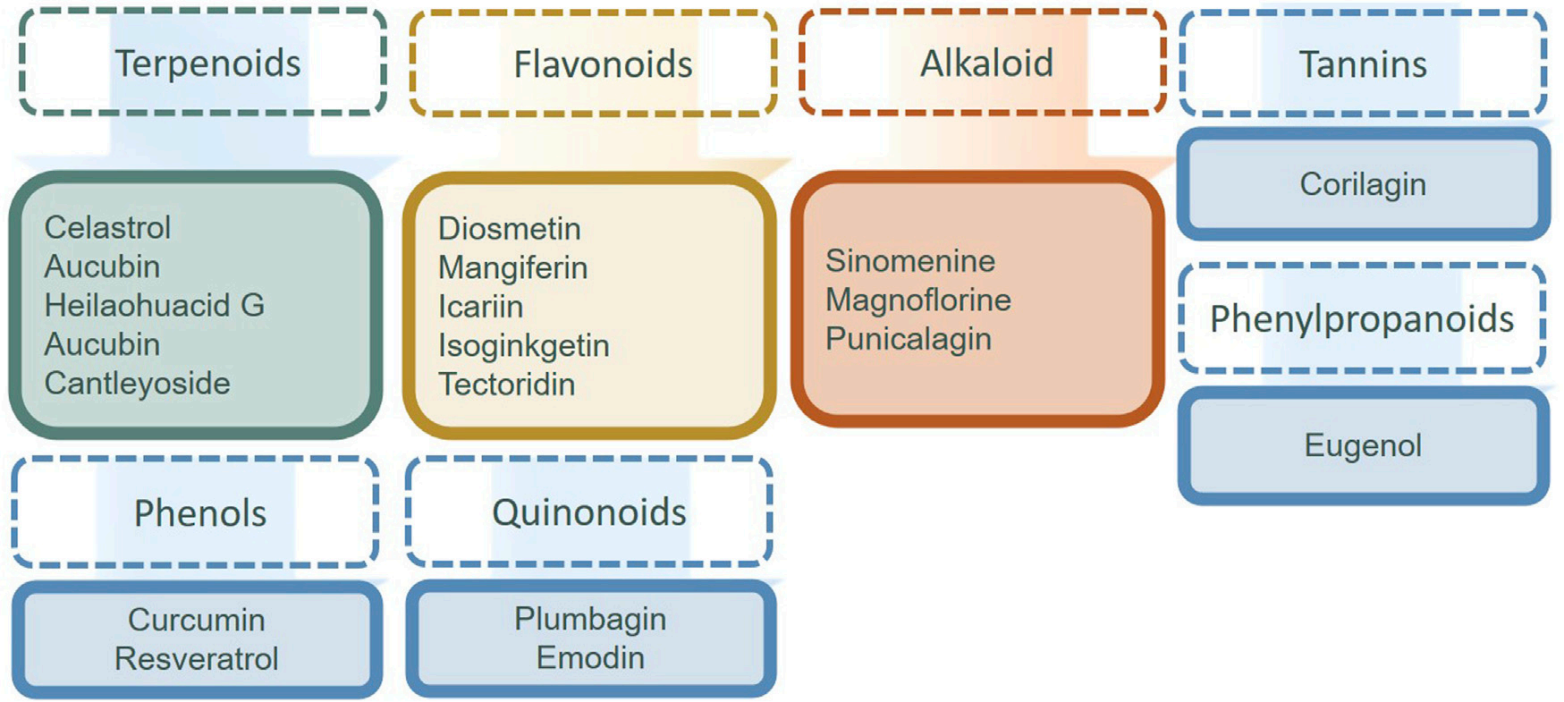
Natural products targeting NF-κB regulating FLS.

The small molecule drugs and natural products targeted at NF-κB in recent 3 years are summarized in Table 2, and the classification of the natural products is in Figure 3. There have been many studies on small molecule compounds that modulate FLS in the NF-κB signaling pathway, such as TAK-242 (Samarpita et al., 2020), CKD-506 (Park et al., 2020), and synthetic derivatives from natural products that also showed the activity of inhibiting proliferation. For example, oxymatrine hydrazone synthesized from oxidized bitter ginseng induced apoptosis and prevented TNF-α-mediated enhanced viability of RA-FLS (Zhang et al., 2021). Paeoniflorin-6′-O-benzene sulfonate (CP-25), a paeoniflorin derivative, had the ability to decrease membrane expression and the combination of these proteins (Wang et al., 2020; Wang et al., 2023). Edaravone, roflumilast, sorafenib, dexmedetomidine, and alogliptin have been used clinically, without the indication for the treatment of RA. The existing experiments showed that they have the anti-proliferation ability of FLS and were worthy of inclusion in the secondary development of drugs. In the natural products in Figure 4, flavonoids still predominated, such as diosmetin, icariin, isoginkgetin, and tectoridin. In a similar situation with the MAPK inhibitions for RA-FLS, these natural products were in the experimental stage. In addition, some inhibitors modulated both NF-κB and MAPK pathways to regulate FLS activity, such as tectoridin and corilagin.

**TABLE 2:** Small molecule drugs targeting NF-κB regulating FLS.

Name	Source	Targets/signaling pathways	Estimate	References
The synthetic small molecule compounds				
TAK-242	A TLR 4 antagonist	TLR4, TLR3; NF-κB	Experimental: TLR4, TLR3 ↓	Samarpita et al. (2020)
			The migration of NF-κB to the nucleus	
			IL-8, IL-1, MMP-7 ↓	
CKD-506	A HDAC inhibitor	NF-κB	Experimental: MMP-1, MMP-3, IL-6, IL-8 ↓	Park et al. (2020)
			The proliferation of Teff ↓	
			Exerting a synergistic effect with MTX	
Oxymatrine hydrazone	Synthesized from oxidized bitter ginseng	MEK/1/2, NF-κB	Experimental: IL-1β, IL-6, IL-8 ↓	Zhang et al. (2021)
			MMP-1, MMP-13 ↓	
			MEK/1/2 and p65 phosphorylation ↓	
Paeoniflorin-6′-O-benzene sulfonate (CP-25)	A paeoniflorin derivative	NF-κB, PI3K, GRK2	Experimental: the protein membrane expression and combination↓	Wang et al. (2020); Wang et al. (2023)
Edaravone	Synthetic: 3-methyl-1-phenyl-2-pyrazolin-5-one	NF-κB, MAPK	Clinical: no indication for the treatment of RA	Zhang et al. (2020); Liu et al. (2023)
			Experimental: altering the antioxidant factors, inflammatory mediators, and pro-inflammatory cytokines [NF-κB, COX-2, and PGE (2)]	
			The level of cytokines and OPN, RANKL, and macrophage M-CSF ↓	
Roflumilast	An inhibitor of phosphodiesterase-4	NF-κB	Clinical: no indication for the treatment of RA	Zhong et al. (2021)
			Experimental: ROS and MDA in MH7A cells ↓	
			IL-6, IL-8, TNF-α↓	
			CCL5, CXCL9, CXCL10 ↓	
			MMP-1, MMP-13 ↓	
Sorafenib	A kinase inhibitor	NF-κB, c-Jun	Clinical: no indication for treatment of RA	Wang et al. (2020)
			Experimental: apoptosis in AA FLSs ↓	
			Fas, caspase-3, Mcl-1 ↑	
			NF-κB, C-Jun ↓	
Dexmedetomidine	A specific and selective alpha-2 adrenoceptor agonist	NF-κB	Clinical: no indication for treatment of RA	Ji et al. (2020)
			Experimental: IL-1β, IL-6, IL-17A, TNF-α, and P-P65↓	
			NLRC5 ↓	
Alogliptin	An important selective dipeptidyl peptidase-4 inhibitor	NF-κB	Clinical: no indication for the treatment of RA	Guo et al. (2020)
			Experimental: MMP-3, MMP-13, IL-6, IL-8, and TNF-α p- Jun, p-IκBβ, nuclear translocation of NF-κB p65 ↓	
The natural products				
Diosmetin	A flavonoid from Rutaceae	NF-κB	Experimental: proliferation of MH7A cells ↓	Chen et al. (2020)
			IL-1β, IL-6, IL-8, MMP-1 ↓	
			and NF-κB pathways activation ↓	
Mangiferin	A flavonoid of the bisphenirone family from mango leaves	ERK2, p38, NF-κB	Experimental: MAPKs (ERK2 and p38), NF-κB ↓	Luczkiewicz et al. (2014); Wang et al. (2021)
Icariin	A flavonoid glycoside from Epimedii Herba	NF-κB	Experimental: TRIB1 ↑ by promoting Nrf2 expression regulating the TRIB1/TLR2/NF-κB pathway	Wu et al. (2022)
Isoginkgetin	A biflavonoid from the leaves of the *Ginkgo biloba* tree	IκBβ, p65	Experimental: IL-1β, IL-6, IL-8 ↓	Shao et al. (2022)
			Migration and invasion of FLS↓ p-IκBα, p-p65, MMP9↓	
Tectoridin	An isoflavone from dry rhizome of iris	TLR4/NLRP3/NF-κB MAPK	Experimental: proliferation of FLS ↓	Huang et al. (2022); Niu et al. (2022)
			Cleaved caspase-3, Bax ↑	
			Bcl-2 ↓	
			Pro-inflammatory cytokines ↓	
			TLR4/NLRP3/NF-κB ↓	
			ERK, JNK, p38 ↓	
Celastrol	A quinone-methylated triterpenoid from *Tripterygium wilfordii*	NF-κB, Notch1, ERK, PI3K/Akt/mTOR	Experimental: NF-кB pathway ↓	Gan et al. (2015); Yu et al. (2015); Doss et al. (2016); Fang et al. (2017); An et al. (2020); Yang et al. (2022)
			NLRP3 inflammasome activation↓	
			ROS ↓	
			Changing some chemokine genes expression (CCL2, CXCL10, CXCL12, CCR2 and CXCR4)	
			SYK-MEK-ERK-NF-κB signaling cascade↓	
			Autophagy ↑	
			PI3K/Akt/mTOR↓	
Aucubin	A monoterpenoid from asterids	NF-κB	Experimental: inflammatory factors ↓	Zhang et al. (2022)
			Bone metabolism factors ↓ p-Iκκ α/β, p-IκBα, p-p65 ↓	
Heilaohuacid G	A triterpenoid from *Kadsura coccinea*/heilaohu	NF-κB	Experimental: apoptosis and inflammatory reactions of FLS↓	Yang et al. (2021); Yang et al. (2022)
Sinomenine	An alkaloid from *Sinomenium acutum*	NF-κB	Experimental: adenosine receptor ↑	Zhou et al. (2017); Yi et al. (2021); Chen et al. (2011); Li et al. (2013); Zhou et al. (2015); Yao et al. (2017)
			NF-κB activation via α7nAChR↓	
			Selective mPGES-1 expression ↓	
			TLR4/MyD88/NF-κB signaling cascade↓	
Magnoflorine	An alkaloid from *Clematis manshurica* Rupr.	PI3K/Akt/NF-κB, Keap1-Nrf2/HO-1	Experimental: proliferation, migration, invasion, and reactive oxygen species levels of MH7A cells ↓	Shen et al. (2022)
			Bax ↑	
			Bcl-2↓ iNOS, COX-2, IL-6, IL-8, MMPs ↓	
			PI3K/Akt/NF-κB ↓	
			Keap1-Nrf2/HO-1 ↑	
Curcumin	A polyphenol from turmeric, *curcuma longa*	NF-κB, AP-1, and p38	Experimental: function of pro-inflammatory mediators↓	Buhrmann et al. (2010); Shang et al. (2016); Mohammadian Haftcheshmeh et al. (2021); Xu et al. (2022)
			Osteoclastogenic potential	
			ERK1/2, p38, JNK ↓	
			RANK, c-Fos, NFATc1 levels↓	
Punicalagin	A polyphenol from pomegranate juice	NF-κB	Experimental: IL-1beta, IL-6, IL-8 and IL-17A ↓	Huang et al. (2021)
			MMP-1 and MMP-13 ↓	
			Proliferation and migration of RA FLSs ↓ phosphorylation of IKK and IkBα ↓	
Corilagin	A tannic acid from *Geranium wilfordii* Maxim.	NF-κB p65, ERK, p38, JNK, IκBα	Experimental: Bcl-2, IL-6, IL-8, MMP-1, MMP-2, MMP-3, MMP-9, COX-2, iNOS ↓	Shen et al. (2022)
			Bax ↑	
			P-p65/p65, P-IκBα/IκBα, P-ERK/ERK, P-JNK/JNK, and P-p38/p38 ↓	
			NF-κB p65 nuclear translocation ↓	
			Proliferation, migration, and invasion of FLS ↓	
Eugenol	A phenylpropanoid from a variety of aromatic herbal plants such as clove and tulsi	NF-κB	Experimental: proliferation, migration, invasion, angiogenesis, and inflammatory response of FLS ↓	Wang et al. (2022)
			NF-κB, COX-2 ↓	
Resveratrol	A phenol from grape	SIRT1, NF-κB	Experimental: SIRT1 and downstream paths ↑	Wang et al. (2020); Sheng et al. (2022)
			The striking interplay between the SIRT1 and NF-κB	
Plumbagin	A naphthoquinone from *Plumbago zeylanica* L.	p65	Experimental: viability of human FLS	Shu et al. (2022)
			Inflammatory cytokines, MMPs ↓	
			IκB, NF-κB, p65 into the nucleus↓	
Emodin	An anthraquinone from rhubarb, buckthorn, etc.	MAPK, NF-κB	Experimental: proliferation of the MH7A cell ↓	Cao et al. (2022)
			MAPK, PTGS2 ↓	
			CASP3↑	
Aucubin	An iridoid glycoside from *Eucommia ulmoides* Oliv.	NF-κB	Experimental: migration and invasion of human FLS ↓	Zhang et al. (2022)
			NF-κB -p65 activity of MC3T3-E1 cells ↓ p-Iκκα β, p-Iκβ, and p-p65 proteins ↓	
Cantleyoside	An iridoid glycoside from *Pterocephalus hookeri* (C. B. Clarke) Hoeck	AMPK/Sirt 1/NF-κB	Experimental: proliferation of human FLS ↓	Bai et al. (2022)
			NO, TNF-α, IL-1β/6, MCP-1 and MMP-1/3/9 ↓	
			OCR, ECAR and real-time ATP generation rate p-NF-κB and translocation ↓	

↓: suppress, downregulate, inhibit, block, prevent, reduce, decrease; ↑: promote, upregulate, active, increase. HDAC, histone deacetylase; PGE (2), prostaglandin E (2); GRK2, G protein-coupled receptor kinase 2; M-CSF, macrophage colony stimulating factor; MDA, malondialdehyde; TRIB1, Tribbles pseudokinase 1; NFATc1, nuclear factor of activated T cells; NLRP3, NOD-like receptor protein 3; HO-1, heme oxygenase; SIRT1, silent information regulator 1; MCP-1, monocyte chemotactic protein-1; OPN, osteopontin; ATP, adenosine triphosphate; α7nAChR, α7 nicotinic acetylcholine receptor; mPGES-1, microsomal prostaglandin E synthase 1; AP-1, activated protein-1.

### 3.3 Small molecule drugs targeting JAK/STAT regulating FLS

JAK/STAT signaling has been instrumental in regulating immune and inflammatory responses. The JAK/STAT pathway can be segmented into three components: receptor-associated tyrosine kinase, JAK tyrosine kinase, and STAT transcription factor. The JAK kinase activates JAK upon receptor binding, leading to JAK-mediated phosphorylation of STAT. Among the STAT family, STAT1 and STAT3 serve as the primary activators (Kim et al., 2011). The expression and activity of STAT1 are elevated in the initial synovial tissue of RA, while STAT3 facilitates the survival of synovial fibroblasts. Elevated STAT3 expression contributes to the inhibition of programmed cell death-induced anti-apoptotic molecule expression, blocks apoptosis in RA-FLS, and promotes RA synovial thickening (Yang et al., 2017). The JAK/STAT pathway is also involved in regulating the response of RA-FLS to pro-inflammatory cytokines and plays an essential role in the pro-inflammatory response and invasive behavior of FLS (Diller et al., 2019).

Inhibitors of JAKs could block the activation of STATs in RA-LS in the synthesis of various drugs and in the study of natural products. We included the synthetic small molecule compounds and natural products in the last 3 years in Table 3. Tofacitinib is a Food and Drug Administration (FDA)- and European Medicines Agency (EMA)-approved JAK inhibitor that effectively treats RA (Vomero et al., 2022). The synthetic small molecule compounds of peficitinib, fingolitinib, takinib, tolvamycin, baricitinib, and abatinib all demonstrated monotherapy effectiveness in clinical trials in RA. The synthetic JAK inhibitors appeared to be an important treatment choice for difficult-to-treat RA patients and researchers (Kubo et al., 2023). Momelotinib had no indication for the treatment of RA in the clinic, but could inhibit the proliferation and migration of FLS (Srivastava et al., 2022). On the contrary, there are few research reports on the natural products in the JAK/STAT signal pathway.

**TABLE 3:** Small molecule drugs targeting JAK/STAT regulating FLS.

Name	Source	Targets/signaling pathways	Estimate	References
The synthetic small molecule compounds				
Peficitinib	A JAK inhibitor	JAK1, JAK2, JAK3, and Tyk2; STAT3	Clinical: phase II and III clinical trials and extension studies completed	Emori et al. (2020); Gutierrez-Urena et al. (2020); Kitanaga et al. (2020)
			Showed efficacy, safety, and tolerability in monotherapy or csDMARDs	
			Experimental: STAT3 phosphorylation by diversified cytokine concentration-dependently ↓	
			Growth factor-A, MMPs, IL-6, TNFSF11 ↓	
Filgotinib	A selective JAK1 inhibitor	JAK1	Clinical: under clinical trial pending approval for use in RA	Shimizu et al. (2023); Westhovens (2023)
			Dose-related effect was not observed for safety excepting for herpes zoster and the increases of lipids and creatine phosphokinase	
Takinib	A selective TAK1 inhibitor	TAK1, TAK3, JNK, NF-κB	Clinical: JAK-STAT pathways in RA patients ↓	Palmroth et al. (2021); Panipinto et al. (2021); Mardani et al. (2023)
			One case of liver failure	
			Experimental: p-TAK1, no effect for the TAK1 downstream factors ↑	
Baricitinib	A JAK 1 and 2 inhibitor	STAT1, JAK	Clinical: monocyte frequency and p-STAT1 in circulating monocytes served as potential early response markers to baricitinib treatment	Tucci et al. (2022); Weston et al. (2022); Taylor et al. (2023)
			Low-risk-related AESI	
			Low incidence with the dermatologic indications	
			Experimental: OSM-induced JAK signaling ↓	
			IL-6, MCP-1, IP-10 expression in the following stages ↓	
Upadacitinib	A selective JAK 1 inhibitor	JAK 1	Clinical: combination with MTX	Panchal et al. (2023); Taldaev et al. (2021)
			Maximum adverse events were reported at 12 mg twice daily	
Tofacitinib	A JAK/STAT inhibitor	STAT6/miR-425-5p/IGF1	Clinical: treatment of RA	Di Benedetto et al. (2021); Palmroth et al. (2021); Panipinto et al. (2021); Liu et al. (2022); Vomero et al. (2022); Ruscitti et al. (2022)
			Beneficial for RA patients who don't respond to TNF-inhibitors or methotrexate	
			Modulate autophagy of FLS	
			Experimental: pro-inflammatory cytokines ↓ collagen I and α-SMA of RA-FLS ↓	
Momelotinib	A competitive JAK1/JAK2 inhibitor	IL-6/JAK1/STAT3	Clinical: no indication for treatment of RA.	Srivastava et al. (2022)
			Experimental: proliferative, migratory of FLS↓	
			PRMT, survivin, HIF-1α ↓	
			JAK1 and STAT3 by IL-6/sIL-6R activation↓	
			SOCS3 ↑	
The natural products				
Matrine	An alkaloid from genus *Sophora*	JAK/STAT; PI3K/Akt/mTOR; TGF-β/Smad; Wnt	Experimental: Bcl-2 ↓	Yang et al. (2017); Ao et al. (2022); Lin et al. (2022)
			Bax, caspase-3↑	
			JAK2, STAT1, STAT3 phosphorylation ↓	
Vitexin	An apigenin flavone glycoside from passion flower, bamboo leaves, and pearl millet	JAK/STAT	Experimental: inflammatory enzyme markers ↓ iNOS ↓	Zhang et al. (2022)
			JAK/STAT expressions ↓	
			SOCS↑	
Isobavachalcone	A chalcone from *Psoralea corylifolia* Linn.	PI3K/Akt, JAK/STAT	Experimental: proliferation, migration, and invasion and promoted apoptosis of MH7A cells ↓ p-PI3K, p-STAT3, p-JAK1 SOCS3, p- Akt ↓	Wang et al. (2022)

↓: suppress, downregulate, inhibit, block, prevent, reduce, decrease; ↑: promote, upregulate, active, increase. csDMARDs, conventional synthetic disease-modifying anti-rheumatic drugs; TNFSF11, TNF Superfamily Member 11; AESI, adverse events of special interest; OSM, oncostatin M; α-SMA, smooth muscle alpha-actin; SOCS, suppressor of cytokine signaling; TAK, TGF β-activated kinase.

### 3.4 Small molecule drugs targeting PI3k/Akt regulating FLS

The PI3K/Akt signaling pathway is involved in regulating cell growth, proliferation, differentiation, and survival and is associated with the production of pro-inflammatory cytokines, degrading enzymes of the extracellular matrix, and other factors in FLS. The activation of PI3K induces the phosphorylation of Akt and p-Akt. As a downstream effector, it can be involved in FLS invasion by regulating the transcriptional levels of MMPs. The Akt phosphorylation also activates downstream mTOR complex 1 (mTORC1). mTORC1 translates mRNA into proteins to regulate the cell activities of metabolism, growth, and differentiation and is involved in RA-FLS proliferation and survival (Wendel et al., 2004; Malemud, 2013).

Table 4 is a summary of the synthetic small molecules and natural drugs that have been developed recently that target PI3k/Akt. Metformin, a drug used to treat type 2 diabetes, has been shown to have a protective effect against the development of RA (Liang et al., 2023), and RA-FLS proliferation is inhibited by metformin in a dose- and time-dependent manner (Chen et al., 2019). The natural products targeted at PI3k/Akt regulating FLS came from a variety of sources. Against the development of inflammatory arthritis, ginger is a preventive substance. There was evidence that ginger helped reduce RA-related joint pain (Al-Nahain et al., 2014). The active ingredients of ginger, 6-shogaol, and 8-shogaol reduced the production of TNF-α, IL-1β, IL-6, etc., prevented migration, invasion, and population growth, and ameliorated joint destruction in mice (N. Li et al., 2023; Jo et al., 2022).

**TABLE 4:** Small molecule drugs targeting PI3k/Akt regulating FLS.

Name	Source	Targets/signaling pathways	Estimate	References
The synthetic small molecule compounds				
Metformin	The biguanide hypoglycemic agents	IGF-IR/PI3K/Akt/m-TOR	Clinical: preventing RA	Liang et al. (2023); Chen et al. (2019); Gharib et al. (2021)
			Inflammation, disease severity, and quality of life with high safety ↑	
			Experimental: G2/M cell cycle phase arrest ↓	
			mTOR phosphorylation ↓	
			Adjusting the p70s6k and 4EBP1 phosphorylation	
The natural products				
Baicalein	A flavone from *Scutellaria baicalensis*	PI3K/Akt/mTOR	Experimental: apoptotic proteins ↑	Zhang et al. (2022)
			EMT-related proteins ↓	
			Cell apoptosis ↑	
			Cell migration phosphorylation ↓	
			The phosphorylation of PI3K, Akt, and mTOR dose dependently ↓	
Nobiletin	A polymethoxylated flavonoid from citrus peels	PI3K/Akt/HIF-1α	Experimental: enhanced the performance in synovial tissue combined with MTX	Liu et al. (2022)
			P-gp expression ↓	
			Contribute to MTX resistance	
Artemisitene	A derivatives of artemisinin from *Artemisia annua* L.	METTL3/ICAM2/PI3K/Akt/p300	Experimental: progression of FLS↓	Chen et al. (2022)
			N6-methyladenosine modification of ICAM2 mRNA ↓	
Shikonin	A naphthoquinone pigment from the root of *Lithospermum erythrorhizon*	PI3K- Akt -mTOR, MAPK	Experimental: migration, adhesion, and invasion of MH7A cells↓	Lian-Hua et al. (2020); Li et al. (2021)
			The phosphorylation levels of Akt, JNK, p38, ERK ↓	
Cinnamaldehyde	An aldehyde from the bark of *Cinnamomum cassia*	PI3K/Akt	Experimental: proliferation and metastasis ↓	Li and Wang (2020)
Daphnetin	A coumarin derivative from *Daphne odora*	PI3K/Akt/mTOR	Experimental: inflammatory response ↓	Deng et al. (2020)
			Cytokine expression ↓	
			IL-10 ↑	
6-Shogaol	An alkylphenol from ginger	PI3K/AKT/NF-κB	Experimental: proliferation, migration, and invasion of FLS and MH7A cells ↓	Li et al. (2023)
			IL-1β, IL-6, IL-8↓	
			MMP-2, MMP-9 ↓	
			PPAR-γ ↑	
8-Shogaol		TAK1, Akt, MAPK	Experimental: TAK1 activity selectively ↓	Jo et al. (2022)
			IKK, Akt, MAPK ↓	
			Reversing pathologies of joint structure	

↓: suppress, downregulate, inhibit, block, prevent, reduce, decrease; ↑: promote, upregulate, active, increase. METTL3, methyltransferase-like 3.

### 3.5 Wnt signaling pathway and relevant drugs regulating FLS

The Wnt signaling cascade participates in regulating the growth, differentiation, production, and apoptosis of osteoblasts. The conventional Wnt/β-catenin cascade, Wnt/Ca_2_^+^ signaling cascade, and Wnt/JNK signaling cascade coordinate with each other to regulate the dynamic balance between osteoclasts and osteoblasts. Once the balance is disturbed, it might lead to bone erosion and bone destruction (Walsh et al., 2009; De, 2011; Deal, 2012). Studies had shown that the growth Wnt3a/5a proteins could activate the Wnt signaling cascade as well as downstream genes, thus increasing fibronectin expression and promoting FLS function. The aforementioned processes also promoted the proliferation of RA synovial tissue without pro-inflammatory factors (Kim et al., 2010; Rabelo Fde et al., 2010; Maeda et al., 2013). Researchers (Cici et al., 2019) suggested that the inflammatory activation of the Wnt pathway might inhibit T-cell function and exacerbate the immune response [181]. In the recent 3 years, we inquired natural products, including paeoniflorin (Yang et al., 2022), 7-hydroxycoumarin (Umbelliferone) (Cai et al., 2022; Cai et al., 2022), and penta-acetyl geniposide (Cai et al., 2021).

## 4 Conclusion

In this review, we summarized as much as possible the involvement of FLS, covering the RA-FLS pathogenesis, synthetic small molecular compounds, and natural products targeting primary signaling pathways in the last 3 years. Natural products comprise a range of substances derived from diverse natural sources, such as plants, animals, and microorganism. These sources provided valuable resources for the design and development of drugs. From the results, the content of this paper could be continuously extended in the following aspects. 1) For the synthetic small molecule compounds, the popular targeting signaling pathways are still MAPK and NF-κB in the current research stage. We cannot ignore that JAK/STAT has great potential for research studies, due to the fact that several drugs have appeared in the clinic. Moreover, modulation of Wnt signaling might not only repair articular bone damage but also inhibit the production of pro-inflammatory cytokines, showing a new strategy for RA treatment (Miao et al., 2013; Liu et al., 2019). Typically, these signaling pathways interacted with each other. A small molecule could act through multiple pathways. 2) For the natural products, there was great potential. Researchers have tried to explore drugs targeted to activate FLS to treat RA using traditional human experience and herbs. For example, triptolide has been a hot area of research for several years. Most of the results are currently in the experimental stage, not the clinical trial stage. Fortunately, the source plants of these natural products have been used for RA in clinical studies. 3) The natural products derived from herbal medicine that can regulate RA-FLS abnormalities are mainly alkaloids, flavonoids, saponins, phenols, and quinones (Smolen et al., 2018). 4) In addition, we have found many reports on the mechanisms of herbal extract, Chinese herbal compound prescription, and traditional Chinese patent medicines in RA that were worthy of further research.

## Figures and Tables

**Table:** 

**RA**	Rheumatoid arthritis
**NFATc1**	c-Fos and nuclear factor of activated T cells c1
**FLS**	Fibroblast-like synoviocyte
**ATF2**	Activating transcription factor-2
**NSAIDs**	Non-steroidal anti-inflammatory drugs
**PGE (2)**	Prostaglandin E (2)
**DMARDs**	Disease-modifying anti-rheumatic drugs
**ROS**	Reactive oxygen species
**MTX**	Methotrexate
**HIF1**	Hypoxia-inducible factor 1
**bDMARDs**	Biologic disease-modifying anti-rheumatic drugs
**CIA**	Collagen-induced arthritis
**ECM**	Extracellular matrix
**IκB**	Inhibitor of κB
**MAPK**	Mitogen-activated protein kinase
**Bcl-2**	B-cell lymphoma-2
**NF-κB**	Nuclear factor kappa-B
**Bax**	Bcl-2-associated X
**JAK**	Janus kinase
**AA**	Adjuvant-induced arthritic
**STAT**	Signal transducers and activators of transcription
**OPG**	Osteoprotegerin
**TNF**	Tumor necrosis factor
**MEKK**	Mitogen-activated protein kinase kinase
**IL**	Interleukin
**IKK**	IκB kinase
**NOS**	Nitric oxide synthase
**TGM2**	Transglutaminase 2
**COX-2**	Cyclooxygenase-2
**NLS**	Nuclear-localization sequence
**RANK**	Receptor activator of NF-κB
**HDAC**	Histone deacetylases
**RANKL**	Receptor activator of NF-κB ligand
**GRK2**	G protein-coupled receptor kinase 2
**VEGF**	Vascular endothelial growth factor
**M-CSF**	Macrophage colony stimulating factor
**MMPs**	Matrix metalloproteinases
**MDA**	Malondialdehyde
**PDGF**	Platelet-derived growth factor
**TRIB1**	Tribbles pseudokinase 1
**TGF-β**	Transforming growth factor-β
**NFATc1**	Nuclear factor of activated T cells
**FLIP**	Anti-apoptotic molecule FLICE inhibitory protein
**NLRP3**	NOD-like receptor protein 3
**TLRs**	Toll-like receptors
**HO-1**	Heme oxygenase
**DKK-1**	Dickkopf-1
**SIRT1**	Silent information regulator 1
**CXCL-8**	CXC motif chemokine 8
**MCP-1**	Monocyte chemotactic protein-1
**CCL2**	CC motif chemokine ligand 2
**OPN**	Osteopontin
**MHCII**	Major histocompatibility complex class II
**ATP**	Adenosine triphosphate
**FcγRs**	Fc-gamma receptors
**α7nAChR**	α7-nicotinic acetylcholine receptor
**APRIL**	A proliferation-inducing ligand
**mPGES-1**	Microsomal prostaglandin E synthase 1
**ABCs**	Age-associated B cells
**AP-1**	Activated protein-1
**VCAM-1**	Vascular cell adhesion molecule-1
**FDA**	Food and Drug Administration
**ICAM-1**	Intercellular cell adhesion molecule-1
**EMA**	European Medicines Agency
**LFA-1**	Lymphocyte function-associated antigen
**csDMARDs**	Conventional synthetic disease-modifying anti-rheumatic drugs
**JNK**	c-Jun N-terminal kinase
**TNFSF11**	TNF Superfamily Member 11
**ERK**	Extracellular regulated protein kinase
**AESI**	Adverse events of special interest
**Bcl-2**	B-cell lymphoma 2
**OSM**	Oncostatin M
**Ang1**	Angiopoietin-1
**α-SMA**	Smooth muscle alpha-actin
**Tie-2**	Tyrosine-protein kinase receptor
**SOCS**	Suppressor of cytokine signaling
**GSK-3**	Glycogen synthase kinase 3
**TAK**	TGF β-activated kinase
**MKK**	Mitogen-activated protein kinase kinase
**mTORC1**	mTOR complex 1
**mTOR**	Mammalian target of rapamycin
